# Green Ambient-Dried Aerogels with a Facile pH-Tunable Surface Charge for Adsorption of Cationic and Anionic Contaminants with High Selectivity

**DOI:** 10.1021/acs.biomac.2c01142

**Published:** 2022-11-01

**Authors:** Zhaleh Atoufi, Goksu Cinar Ciftci, Michael S. Reid, Per A. Larsson, Lars Wågberg

**Affiliations:** † Department of Fiber and Polymer Technology, KTH Royal Institute of Technology, Teknikringen 56−58, SE-100 44Stockholm, Sweden; ‡ Department of Fiber and Polymer Technology, Wallenberg Wood Science Center (WWSC), KTH Royal Institute of Technology, SE-100 44Stockholm, Sweden

## Abstract

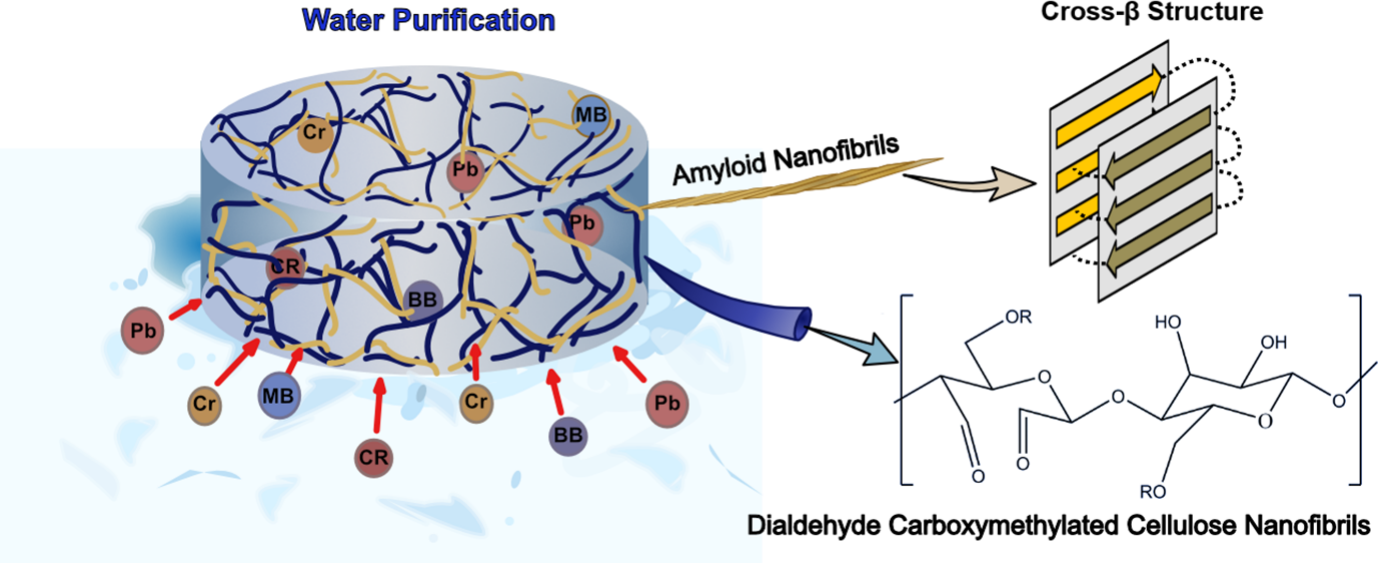

The fabrication of reusable, sustainable adsorbents from low-cost, renewable resources via energy efficient methods is challenging. This paper presents wet-stable, carboxymethylated cellulose nanofibril (CNF) and amyloid nanofibril (ANF) based aerogel-like adsorbents prepared through efficient and green processes for the removal of metal ions and dyes from water. The aerogels exhibit tunable densities (18–28 kg m^–3^), wet resilience, and an interconnected porous structure (99% porosity), with a pH controllable surface charge for adsorption of both cationic (methylene blue and Pb(II)) and anionic (brilliant blue, congo red, and Cr(VI)) model contaminants. The Langmuir saturation adsorption capacity of the aerogel was calculated to be 68, 79, and 42 mg g^–1^ for brilliant blue, Pb(II), and Cr(VI), respectively. Adsorption kinetic studies for the adsorption of brilliant blue as a model contaminant demonstrated that a pseudo-second-order model best fitted the experimental data and that an intraparticle diffusion model suggests that there are three adsorption stages in the adsorption of brilliant blue on the aerogel. Following three cycles of adsorption and regeneration, the aerogels maintained nearly 97 and 96% of their adsorption capacity for methylene blue and Pb(II) as cationic contaminants and 89 and 80% for brilliant blue and Cr(VI) as anionic contaminants. Moreover, the aerogels showed remarkable selectivity for Pb(II) in the presence of calcium and magnesium as background ions, with a selectivity coefficient more than 2 orders of magnitude higher than calcium and magnesium. Overall, the energy-efficient and sustainable fabrication procedure, along with good structural stability, reusability, and selectivity, makes these aerogels very promising for water purification applications.

## 1 Introduction

The lack of access to clean water has become an acute environmental challenge facing rural populations in recent decades. It has become such a global issue that access to water and sanitation is goal number six of the United Nations’ sustainable development goals.^1^ In many countries, untreated industrial discharges from textile, cosmetics, mining, and alloying processes are the main source of contaminants. Organic dyes such as congo red (CR), brilliant blue (BB), and methylene blue (MB) are intrinsically harmful to human health and ecosystems.^2,3^ These dyes are highly water soluble and are difficult to degrade due to their chemical and thermal stability.^4,5^ In addition to dyes, metal ions are other water contaminants that cause both acute and chronic toxicity in aqueous environments. Among them, hexavalent chromium (Cr(VI)) is a well-known genotoxic and mutagenic pollutant that significantly threatens human and aquatic organism health.^6^ Moreover, Cr(VI) is 100 times more toxic^7^ and 500 times more soluble in aqueous media than Cr(III).^8^ Another life-threatening, common metal ion in water is Pb(II), which is well known to cause severe neurotoxicity and carcinogenicity and can damage the digestive, urinary, and central nervous systems.^9^ According to the United States Environmental Protection Agency, the permissible total amounts of Cr and Pb in drinking water is below 0.1 and 0.015 mg L^–1^, respectively.^10^ Therefore, different technologies have been proposed for the decontamination of these pollutants including membrane separation,^11^ chemical precipitation,^12^ biodegradation,^13^ photodegradation,^14^ and adsorption.^15,16^ Among these methods, adsorption is recognized as one of the most promising approaches owing to its low cost, simple implementation, and the absence of secondary pollutants.^2^ It has been reported that the cost of water treatment with nonadsorption technologies is in the range of 10 to 450 USD/m^3^ of purified water, whereas adsorption-based approaches range from 5 to 200 USD/m^3^ with approximately 70% of the cost related to the production of the adsorbent material.^17^ As a result, the development of energy-efficient methods to prepare adsorbents from low-cost and biobased materials is potentially a viable and sustainable approach for water purification.

Aerogels, being ultralight highly porous materials with high specific surface areas, are attractive structures for adsorption applications.^18^ Considering the importance of environmental protection and sustainable development, preparation of aerogels from natural renewable recourses is vital for obtaining sustainable and yet efficient sorbents. Recent state-of-the-art research on amyloid nanofibrils (ANFs) has spurred the development of amyloid-based functional materials for the adsorption of metal ions and organic pollutants. ANFs consist of several amino acid residues providing multivalent binding to organic pollutants and metal ions.^19^ ANFs can be prepared from a large number of different proteins, including the edible milk protein β-lactoglobulin. β-Lactoglobulin can be obtained from whey, which is cheap and highly available as a byproduct of the cheese-making process in the dairy industry.^19^ Under controlled conditions, β-lactoglobulin can form long, multistranded, semiflexible ANFs with remarkable structural stability and mechanical properties due to their rigid cross-β sheet core structure.^20,21^ As a result of their high aspect ratio, these long and slender ANFs are capable of forming stable gel networks at low solid contents, and the gel-to-solid transition of such a gel system can be used for making multifunctional amyloid-based aerogels. Although the potential of aerogels from ANF in the remediation of environmental pollutants has been shown in different studies,^22−24^ the material preparation most frequently requires time- and energy-demanding drying processes (such as freeze-drying or supercritical drying) to prevent shrinkage, cracking, and collapse of the ANF-based aerogels during fabrication.

Another biosourced nanomaterial that has frequently been used for making lightweight materials is the cellulose nanofibril (CNF).^25^ CNFs not only have outstanding physical properties, including high aspect ratio and high elastic modulus, but they can also be functionalized with different chemical moieties to increase their interactions with metal ions and dyes.^26^ For example, partial carboxymethylation is a commonly applied pretreatment method to prepare highly charged CNFs and thereby improve the CNFs’ affinity to metal ions.^27^ In addition, CNF-based aerogel sorbents can be fabricated via a simple and scalable freeze-linking method without the need for hazardous chemical cross-linkers, harmful solvents, and energy-consuming freeze-drying methods.^28^ In this procedure, a dispersion of CNFs is initially frozen under regular freezer conditions, i.e., around −20 °C, whereby the growth of the ice crystals squeezes the CNFs together to form an interconnected porous structure with thick and strong fibrous pore walls. Subsequently, the frozen structure is thawed and solvent exchanged to acetone to reduce the capillary forces to preserve the aerogel-like structure during ambient drying conditions. By performing a periodate oxidation pretreatment on the CNFs prior to aerogel fabrication, aldehyde groups are introduced on the CNFs, and these can form hemiacetal linkages during ice templating, providing structural stability in wet conditions.^28,29^

We hypothesized that by controlled mixtures of dialdehyde carboxymethylated cellulose nanofibrils (DA-C-CNFs) and ANFs, ambient-dried wet-stable aerogel-like adsorbents can be prepared using the freeze linking methodology. The main role of DA-C-CNFs was to provide wet stability by cross-linking the aerogels, while ANFs were used due to their amphoteric nature that enabled the aerogels to interact with both cationic and anionic contaminants. The chemical structure, thermal stability, microstructure, and pore-size distribution of the aerogels were characterized, and the homogeneity of the structure was established using confocal microscopy. Following this, the adsorption potential of the aerogels for two anionic dyes (BB and CR), one cationic dye (MB), and two metal ions (Cr(VI) and Pb(II)) at different pHs was investigated. Adsorption isotherms were collected to determine the adsorption capacity of the aerogel under different equilibrium concentrations of model contaminants to study the fundamental interactions between the adsorbent and adsorbate. The adsorption kinetics was also investigated, and the reusability of the aerogel was assessed by cyclic sorption–desorption tests. Finally, the selectivity of the aerogel for Pb(II) in the presence of background ions was tested. Overall, the hybrid aerogels showed a very promising potential in water purification applications.

## 2 Materials and Methods

### 2.1 Materials

Carboxymethylated CNFs (C-CNFs) in the form of a 2.2 wt % aqueous gel with a total charge of 600 ± 50 μequiv g^–1^ were produced according to a previously reported method^30^ and provided by RISE Bioeconomy AB. Lyophilized powder of β-lactoglobulin from bovine milk (≥90% purity) was purchased from Merck. Thioflavin T (ThT), BB, MB, CR, sodium hydroxide, Triton X-100 solution (10 wt % in water), lead acetate trihydrate, chromium(VI) oxide, and hydrochloric acid solution of 37 wt % were purchased from Merck. Sodium metaperiodate (99%) was purchased from Acros Organic, Belgium. Acetone and ethanol were purchased from VWR International (Radnor, PA, USA). All materials were used without further purification.

### 2.2 Preparation of DA-C-CNF

DA-C-CNF was prepared by periodate oxidation of C-CNFs according to a previously reported method.^29^ Briefly, C-CNFs were diluted with Milli-Q water to a concentration of 1.5 wt %. Sodium metaperiodate was added to the C-CNF dispersion to reach a final concentration of 60 mM and mixed with an Ultra Turrax (IKA Werke GmbH & Co. KG, Staufen, Germany) at 12,000 rpm for 5 min. The oxidation reaction was then continued for 2 h by incubating the dispersion in the dark to avoid the photodegradation of periodates.^31^ Finally, the reaction was quenched by adding a stoichiometric excess of ethylene glycol. The oxidized C-CNFs were then dialyzed for 1 week under continuous exchange of DI water to remove all the unreacted reagents. The obtained DA-C-CNFs were stored in a refrigerator for further use.

### 2.3 Preparation of ANFs

ANFs were prepared from denaturation and self-assembly of β-lactoglobulin proteins.^32^ A 2 wt % solution of β-lactoglobulin was prepared, and the pH was adjusted to pH 2 using a 1 M HCl solution. The solution was then incubated at 90 °C for 5 h under mild stirring. During this process, the proteins unfold, hydrolyze, and reassemble into a cross-β-sheet structure to form amyloid nanofibrils.^33^

### 2.4 Aerogel Preparation Procedure

DA-C-CNF (pH 6) and ANF (pH 2) gels were diluted to a concentration of 1 wt % and mixed by magnetic stirring at three DA-C-CNF/ANF ratios: 60:40, 50:50, and 40:60. The gel mixtures were then cast into polystyrene Petri dishes with a diameter of 30 mm and frozen at −18 °C for 4 h. The frozen samples were thawed by soaking the molds in ethanol for 20 min. Once the samples were entirely thawed, they were detached from the molds and solvent exchanged to acetone by submerging them into acetone and replacing the acetone with fresh acetone every 15 min three times. Finally, the samples were dried under ambient conditions. Samples with higher densities were prepared via the same procedure but with the concentration of the initial gels adjusted to values of 1.25, 1.5, and 1.7 wt %.

### 2.5 Carbonyl Content Determination

The aldehyde content of the DA-C-CNFs was estimated using a titration method described earlier.^28,34^ Briefly, 0.1 g of DA-C-CNFs was added to 25 mL of a 10 mM NaCl solution, and the pH was adjusted to 4. Then, 25 mL of a 0.25 M solution of hydroxylamine hydrochloride in 10 mM NaCl, adjusted to pH 4, was added to the DA-C-CNF mixture and stirred for 2 h to allow for a reaction between hydroxylamine hydrochloride and all the available aldehyde groups, releasing a stoichiometric number of protons. After 2 h, the mixture was titrated back to pH 4 using a 0.1 M NaOH solution, and the aldehyde content of DA-C-CNF was calculated from the moles of NaOH required. Samples were tested in triplicate.

### 2.6 Density and Porosity Measurements

The weight of the aerogels was measured gravimetrically using an analytical balance, and the volume was calculated by careful measurement of the dimensions using a caliper. The porosity was calculated according to the following:
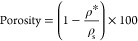
1where ρ* is the apparent density of the aerogels calculated by dividing the mass of aerogels to their volume. ρ_s_ is the pore wall density^35^ that was estimated according to the ″rule-of-mixture″ equation:
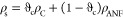
2where ρ_c_ is the density of DA-C-CNFs, υ_c_ is the volume fraction of DA-C-CNFs in the aerogel, and ρ_ANF_ is the density of ANFs. ρ_c_ was estimated to be the cellulose density (1500 kg m^–3^),^36^ and ρ_ANF_, the density of ANFs, was taken as 1300 kg m^–3^.^37^

### 2.7 Automated Pore Volume Distribution Measurement

The pore volume distribution of the aerogels was measured using a TRI Autoporosimeter (APVD) version 2008–12 (Princeton, USA) with a membrane cutoff radius of 1.2 μm. Aerogels were wetted in a 0.05 g L^–1^ solution of Triton X-100 in water and placed in the closed chamber of the instrument where a gradual increment of the air pressure led to a consecutive depleting of the pores. By measuring the amount of discharged water at each pressure step, cumulative pore volume distribution was obtained. In this study, 10 different pressure steps corresponding to pore radius in the range of 5 to 500 μm were used. The pore radius corresponding to the chamber gas pressure was calculated according to eq 3:
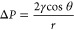
3where Δ*P* is the pressure difference between the applied chamber pressure and atmospheric pressure; γ is the liquid–gas surface tension, in this case 30 mN m^–1^ for Triton X solution and air; θ is the receding solid–liquid contact angle (assuming that the surface is fully wetted by using Triton X as surfactant, cos θ = 1); and *r* is the pore radius.

### 2.8 Mechanical Testing

The compressive mechanical properties of the aerogels in the dry and wet state were assessed using an Instron 5566 universal testing machine (Norwood, USA) equipped with a 500 N load cell and cylindrical samples with a diameter of approximately 30 mm and a thickness of 10 mm. Dry compressive tests were carried out by compressing the aerogels up to 70% with a strain rate of 10%/min at a controlled temperature (23 °C) and humidity (50%). For the wet compression test, aerogels were soaked in water for 24 h prior to the test. All tests were performed in triplicate.

### 2.9 Thermogravimetric Analysis

The thermal stability of the aerogels was assessed by thermogravimetric analysis (TGA) using a Mettler Toledo TGA/DSC 1 STARe System. Samples were heated from 30 to 700 °C at a heating rate of 10 °C/min in a nitrogen atmosphere. The specimen weight was approximately 4 mg, and specimens were conditioned at room temperature for 8 h before measurement.

### 2.10 Scanning Electron Microscopy

Scanning electron microscopy (SEM) was performed using an S-4800 field emission scanning electron microscope (Hitachi, Tokyo, Japan) at a 4 kV accelerating voltage. Aerogels were soaked in liquid nitrogen for 2 min, cut into very thin layers using a sharp razor blade, and glued on a sample holder using carbon tape. The samples were then sputter-coated with approximately 4 nm of Pt–Pd alloy using a Cressington 208 HR sputter coater (Cressington Scientific Instruments, Watford, UK).

### 2.11 Confocal Microscopy

To perform confocal microscopy, ANFs were stained by ThT as an amyloid binding fluorescent dye. A stock solution of 5 mM ThT in PBS buffer (pH 7) was prepared, and 0.1 mL of it was added to 9.9 mL of a 2 wt % ANF dispersion to reach a total ThT concentration of 50 μM. The dispersion was stirred for 5 h to let the ThT bond to the ANFs and then dialyzed in the dark to remove nonbonded ThT. The dialysate was replaced with a fresh solution three times a day, and the absorption spectrum of the dialysate was collected using a plate reader. The absorption spectrum of free ThT in the water solution shows a peak at λmax = 412, and the dialysis process was continued until no absorption signal was detected from ThT. The ThT-stained ANFs were then utilized to prepare aerogels for confocal microscopy. Aerogels were prepared by ice-templating as explained in detail before but dried via freeze-drying instead of solvent exchange to eliminate the possible effects that acetone might have on the ThT. Confocal microscopy was performed using a Confocal Microscope Zeiss LSM 800 Airyscan with a Plan-Apo 63×/1.40 NA Oil objective. The samples were excited using a 458 nm argon laser, and the emission was collected between 480 and 520 nm. Images were analyzed using the ZEN 3.4 software.

### 2.12 Zeta Potential Measurements

The zeta potential of the ANFs and the DA-C-CNFs in the pH range of 2.5–10.5 and 25 °C was analyzed using a dynamic light scattering instrument (Zetasizer Pro, Malvern) equipped with an MPT-3 Multi-purpose Titrator. DA-C-CNFs and ANFs were diluted to 0.1 wt %, and the pH was adjusted using 0.1 and 0.25 M NaOH solution and 0.25 M HCl solution, respectively.

### 2.13 Atomic Force Microscopy

ANF dispersion was diluted to 0.005 wt % with Milli-Q water and drop cast on a clean silicon oxide substrate. The samples were then imaged using a MultiMode 8 atomic force microscope (AFM, Bruker, Santa Barbara, CA) in TappingMode with RTESP-150 cantilevers. Images were analyzed using the NanoScope Analysis software.

### 2.14 Adsorption Experiments

A series of batch adsorption experiments were conducted to determine the adsorption behavior of the aerogels for different dyes and metal ions. Initially, stock solutions of CR, BB, MB, Cr(VI), and Pb(II) with a concentration of 300 mg.L^–1^ were prepared and diluted to the desired concentration with Milli-Q water. To measure the adsorption capacity of aerogels at different pHs, aerogels were cut into small pieces of approximately 15 mg and submerged into 15 mL solutions of 50 mg/L BB, MB, Cr(VI), and Pb(II) and 100 mg/L CR. The pH of the dye solutions was adjusted to 4, 6, and 8.5 and the pH of metal solutions was adjusted to 2.5, 4, and 5.8 using 0.1 M HCl and NaOH solutions. Higher pHs were not tested for metal ions due to the hydrolysis and precipitation of Pb at pHs above 6. After 48 h of incubation (to ensure that equilibrium was reached), 150 μL of the dye solution was taken from the vial, and the dye concentration was tested using a Tecan Infinite 200 PRO plate reader, Switzerland. The calibration curves of the dyes are shown in Figure S1. The concentration of metal ions was tested by ALS-Scandinavia, in Luleå, Sweden, using an inductively coupled plasma sector field mass spectrometry (ICP-SFMS) Element 1, Finnigan MAT instrument. All samples were acidified with 1% HNO_3_ and later diluted prior to analysis. The adsorption capacity of the aerogels was then calculated according to eq 4:
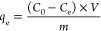
4where *q*_e_ (mg/g), *C*_0_ (mg/L), *C*_e_ (mg/L), *V* (L), and *m* (g) are the equilibrium adsorption capacity, initial concentration, equilibrium concentration, volume of contaminant solution, and mass of aerogel, respectively.^38^ The kinetics of sorption was assessed by incubating 15 mg of aerogel in 15 mL of 100 mg L^–1^ solution of BB at pH 6 followed by taking 150 μL of the dye solution at specific time intervals. An average of three measurements is reported. Kinetics data were then fitted to pseudo-first-order (eq 5), pseudo-second-order (eq 6), and intraparticle diffusion models (eq 7):
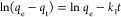
5
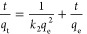
6
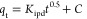
7where *q*_t_ (mg g^–1^) is the adsorption capacity of the aerogels at time *t*; *k*_1_, *k*_2_, and *K*_ipd_ are the adsorption rate constants of the pseudo-first-order, pseudo-second-order, and intraparticle diffusion model, respectively; and *C* is a constant for the experiment.

To determine the adsorption behavior of the aerogel for BB as a model dye and Cr(VI) and Pb(II), approximately 15 mg of the sample was submerged in 25 mL of the contaminant solutions of different concentrations (ranging from 5 to 300 mg/L) at pH 6 and shaken for 48 h on a shaking board. The resulting adsorption data were fit to Langmuir (eq 8) and Freundlich (eq 9) isotherm models:
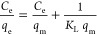
8
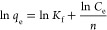
9where *q*_m_ (mg g^–1^) is the maximum adsorption capacity of the aerogels; *K*_L_ and *K*_f_ are the binding constant of the Langmuir and Freundlich equation, respectively; and 1/*n* is an empirical constant.

### 2.15 Reusability and Selectivity

The reusability of the aerogels was studied for BB and Cr(VI) as anionic model contaminants and MB and Pb(II) as cationic model contaminants. First, the aerogels were soaked in an ethanol solution containing 0.1 M HCl for 1 h to transform hemiacetal linkages to pH-stable acetal linkages that provide stability for the aerogels at higher pHs required for the regeneration process. The treated aerogels were then washed with ethanol and dried at ambient conditions. To test the reusability, approximately 90 mg of pretreated aerogel samples was soaked in 100 mL of the contaminant solution with a concentration of 30 mg L^–1^ for 6 h. For each contaminant, the pH was adjusted to the earlier determined optimum pH (i.e., pH 2.5 for Cr, pH 4 for BB, pH 5.8 for Pb, and pH 8.5 for Mb) prior to the test. After adsorption, the cationic contaminants were desorbed by soaking the aerogels in a 0.01 HCl solution, and anionic contaminants were desorbed by soaking the aerogels in a 0.01 M NaOH solution. The aerogels were then washed with water, solvent exchanged to acetone, and dried ambiently for the next adsorption–desorption cycle. Three cycles of sorption and desorption were performed, and an average of three experiments is reported.

To test the selective removal of Pb(II) in aqueous media containing other background ions, samples of approximately 14 mg of aerogel were soaked in 25 mL solutions containing Pb(II), Ca(II), and Mg(II) ions with a concentration of 14 mg/L for each metal ion. After 48 h, the remaining concentration of each metal ion in the solution was measured by ICP-SFMS, and the removal efficiency was calculated. The distribution coefficient (*K*_d_) and selectivity coefficient (α) were calculated by the following equations:
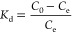
10
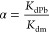
11where *C*_0_ and *C*_e_ are the initial and equilibrium concentration of metal ions in the solution. *K*_dPb_ and *K*_dm_ are the distribution coefficient of Pb(II) and the background ions in the solution, respectively.^39^

## 3 Results and Discussion

### 3.1 Principles of Aerogel Preparations

ANFs formed by heating a β-lactoglobulin protein solution at 90 °C and pH 2 exhibited the generic morphology of semiflexible amyloid nanofibrils.^21^ According to AFM images, ANFs had a high aspect ratio of approximately 1070 (average length of 4.9 μm and diameter of 4.5 nm) (Figure S2). The high aspect ratio enables the fibrils to form self-supporting physically entangled networks at low concentrations, which are a desired characteristic for forming robust aerogels.^40^ The second biocomponent, DA-C-CNF with an aldehyde content of 1.0 mmol g^–1^, was successfully produced via periodate oxidation of C-CNFs with an aspect ratio of approximately 200.^40^ Aerogels were prepared by mixing the dispersions at three different ratios of DA-C-CNFs to ANFs (60:40, 50:50, and 40:60) followed by freezing in a conventional freezer (−18 °C), thawing, solvent exchange, and ambient drying (Table 1).

**Table 1:** Composition and Physical Properties of the Biohybrid Aerogels^a^

	composition (%)				
sample	DA-C-CNFs	ANFs	density (kg/m^3^)	porosity (%)	wet shape recovery (%)
DA-C-CNF/ANF 60:40	60	40	18	98.7	66.1
DA-C-CNF/ANF 50:50	50	50	16	98.8	40.9
DA-C-CNF/ANF 40:60	40	60			

a In the text, the term aerogel specifically refers to DA-C-CNF/ANF 60:40 as it has the optimum properties. Otherwise, the name of the sample is mentioned explicitly.

**Figure 1:**
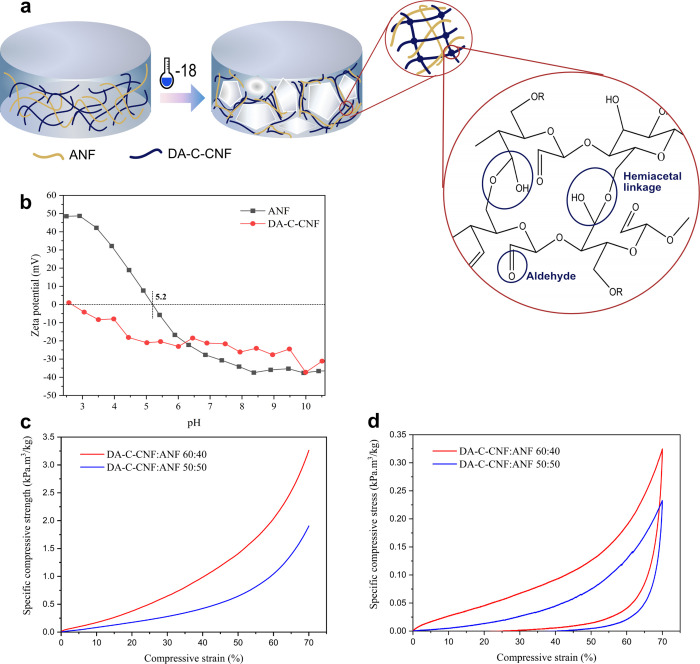
(a) Schematic illustration of the formation of a porous structure via ice templating. During freezing, the growth of ice crystals pushes the nanofibrils together to form a highly packed fibrillar lamellar structure where aldehyde groups on DA-C-CNFs form hemiacetal linkages and cross-link the structure. (b) Zeta potential of ANFs and DA-C-CNFs at various pHs. ANF isoelectric point is 5.2. Compressive stress–strain curve of biohybrid aerogels of different compositions in the (c) dry and (d) wet state.

To form a porous material that can withstand ambient drying without structural collapse, a strong interconnected network is required. As stated earlier, the DA-C-CNFs and ANFs dispersions were oppositely charged having zeta potentials of −23 and 48 mV (Figure 1b), respectively. Upon mixing the dispersions, biohybrid associates were formed due to the electrostatic interactions. During the freezing process, these associates were excluded from the growing ice crystals and packed in thin lamellae between the ice crystals. Consequently, the distance between DA-C-CNFs in the lamellae decreased, and the aldehyde groups of DA-C-CNFs reacted with the surrounding alcohol groups on the cellulose backbone, forming hemiacetal linkages.^29^ This cross-linked network of DA-C-CNFs acted as the main skeleton that maintained the integrity of the structure, while ANFs were locked into the network due to the entanglements and charge-driven interactions. This mechanism is schematically shown in Figure 1a. The lamellae structure in the aerogels formed with DA-C-CNF/ANF ratios of 60:40 and 50:50 were strong enough to resist the thawing process and the stresses imposed by capillary forces during the drying from acetone. In contrast, samples with a DA-C-CNF/ANF ratio of 40:60 disintegrated during the thawing step. A possible reason for this is that the ANFs prevented the close contact between the DA-C-CNFs needed to form hemiacetal cross-links at this fibril ratio. Moreover, this also clearly demonstrates that the noncovalent interactions between ANFs and DA-C-CNFs were not strong enough to preserve the porous structure during thawing and drying.

The wet and dry mechanical properties of the aerogels were assessed as these characteristics are essential for water purification and many other advanced applications, including tissue engineering and energy storage devices with liquid electrolytes. The compression results show that the dry specific modulus of DA-C-CNF/ANF 60:40 was 2.6 times higher than that of DA-C-CNF/ANF 50:50 (Figure 1c). Moreover, in the wet state, the 60:40 aerogels could recover up to 66% of their initial shape, while the 50:50 aerogels could recover only 41% of their shape after 70% compression (Figure 1d). These results indicate that the higher cross-linking of DA-C-CNF/ANF 60:40 leads to a stiffer structure. As a result, DA-C-CNF/ANF 60:40 was chosen as the optimum structure for further characterizations, and hereafter, the term aerogel refers specifically to DA-C-CNF/ANF 60:40. At this ratio, aerogel densities were tuned from 18 to 27 kg m^–3^ by changing the initial concentrations of DA-C-CNF and ANF dispersions (Figure S3). A picture of the aerogel with the lowest density is also shown in Figure S3.

### 3.2 Chemical and Structural Characterizations

**Figure 2:**
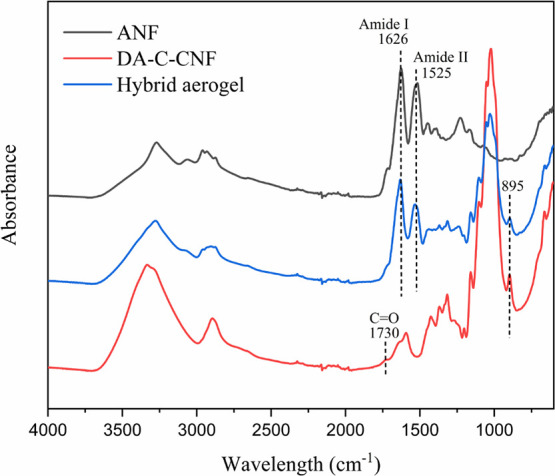
FTIR spectra of ANFs, DA-C-CNFs, and the biohybrid aerogel of DA-C-CNF/ANF.

The chemical composition of ANFs, DA-C-CNFs, and the hybrid aerogel was characterized by FTIR. As shown in Figure 2, the spectrum of hybrid aerogel contains the characteristic signals of ANFs and DA-C-CNFs. DA-C-CNFs showed the characteristic cellulose peaks ranging from 915 to 1180 cm^–1^ attributed to the C–O stretching vibration signals of primary alcohols and intra- and extracyclic C–O of O–C–O–C groups in the cellulose backbone.^41^ The broad peak between 3000 and 3650 cm^–1^ is assigned to the O–H and C–H stretching vibration. The peak at 1592 cm^–1^ is attributed to the asymmetric stretching vibrations of carboxylic groups, and the weak peak at 1730 cm^–1^ is attributed to the vibration of C=O bands of the aldehyde groups.^42,43^ Hemiacetals that should be present, considering the wet stability of DA-C-CNF, usually show a vibration band at nearly 885 cm^–1^, and this signal is most probably masked by the signal at 895 cm^–1^ attributed to the in-plane symmetric vibration of β-glycosidic linkages between the glucose units of CNFs.^44,45^ Nonetheless, hemiacetal linkages have been shown to be present in freeze-linked CNF aerogels despite the lack of signal in FTIR.^29^ ANFs displayed the characteristic amide I and amide II peak at 1626 and 1525 cm^–1^ that are in agreement with the reported values for β-lactoglobulin derived amyloid fibrils.^46^ Moreover, the broad peak between 3000 and 3600 cm^–1^ is due to the stretch vibration of O–H and N–H groups.^47^

**Figure 3:**
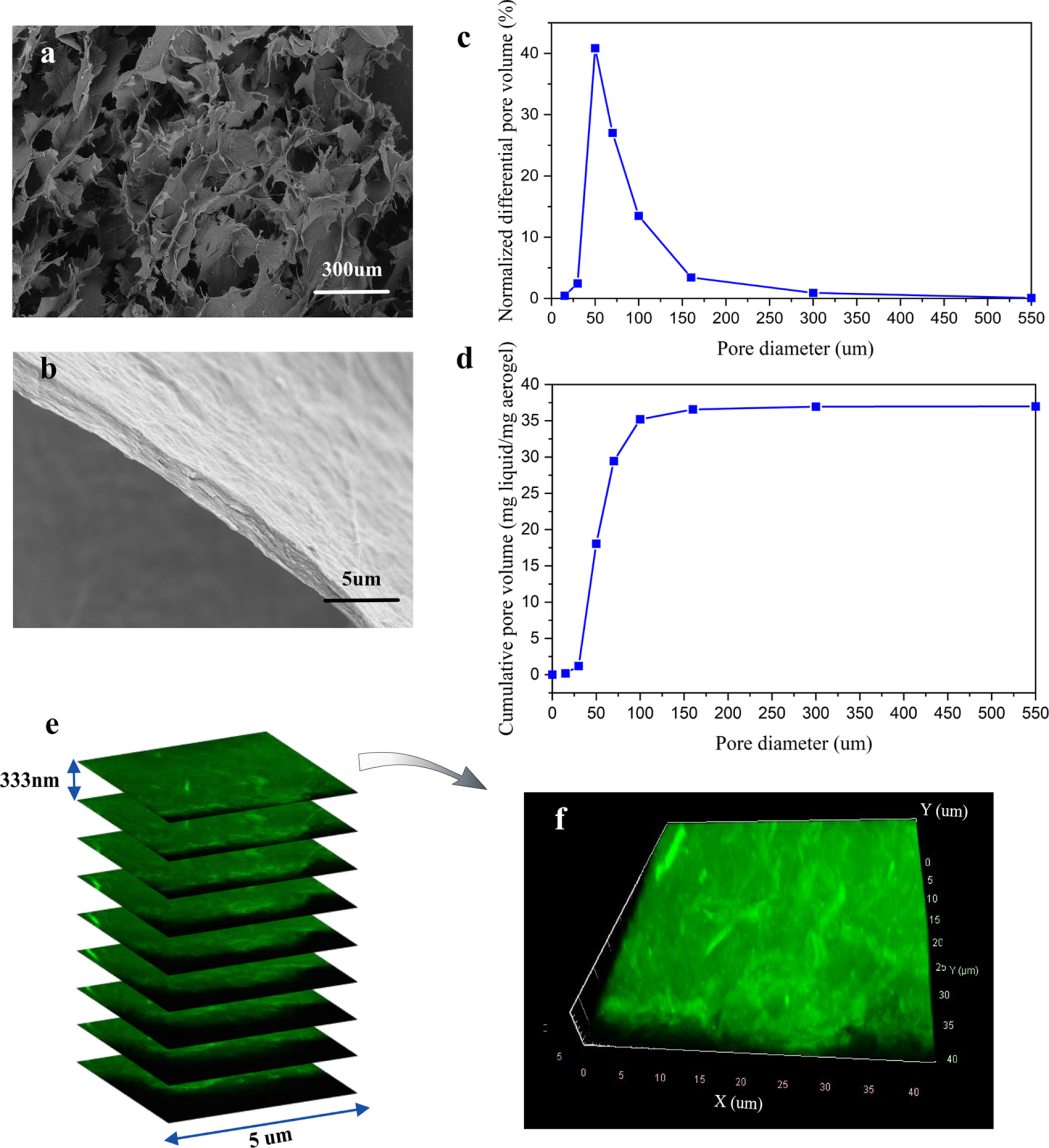
Structural characteristics of a DA-C-CNF/ANF aerogel. SEM image of (a) the porous structure and (b) lamellar pore walls of the aerogels. (c) Normalized differential pore volume distributions of the aerogel. (d) Confocal images taken from the aerogel lamella *Z*-stacks. (e) 3D image generated by of the individual *Z*-stacks.

The microstructure of the aerogel shows a characteristic ice-templated porous structure with pores ranging from tens to hundreds of micrometers, similar to the previously reported results for ice-templated networks (Figure 3a).^28^ The pore walls, formed during ice crystal growth, have a packed, lamellar structure consisting of DA-C-CNF/ANF associates (Figure 3b). By soaking the aerogels in water, they absorbed water up to 88% of their bulk volume and became almost transparent, which demonstrates the open porous structure of the aerogels as inaccessible air pockets would scatter light and make the aerogels opaque. The water wet pore size distribution of the aerogel was measured by APVD, and the pore volume distribution and the cumulative pore volume results (Figure 3c,d) quantify and support the SEM images showing a polydisperse pore size distribution with the majority of pores ranging from 30 to 300 μm, where 41% of pore volumes belong to the pores with an average diameter of 50 μm. Moreover, from the APVD measurements, it is shown that 14% of the total volume can be found in pores smaller than 15 μm in diameter. Although it is not possible to probe the pore sizes smaller than 10 μm with APVD, this value was determined by measuring the volume of water that remained in the smaller pores by weighing the aerogels before and after the test,^48^ which is probably the water retained in the swollen structure of lamellae.

To investigate the distribution of ANFs in the aerogel structure with confocal microscopy, ANFs were labeled with ThT, a dye molecule that exhibits enhanced fluorescent signal upon binding to ANFs, with excitation and emission maxima at approximately 450 and 483 nm, respectively.^49^ The hundred-fold increase in the ThT fluorescent intensity upon binding to ANFs is assigned to the rotational immobilization of the carbon–carbon bond connecting the benzylamine and benzothiazole rings.^50^ Distribution of the ANFs at the bulk of the aerogels was assessed by taking confocal images at different sample depths using the *Z*-stack function. Figure 3e,f shows the acquired Z-stacks from an aerogel lamella and the rendered 3D image showing the homogeneous distribution of ANF bundles throughout the hybrid aerogel. The existence of larger associates of ANFs, compared to the AFM images of the ANFs shown in Figure S2, also indicates that there has been an association but no macroscopic flocculation between the ANFs and the DA-C-CNFs.

### 3.3 Thermal Properties of the Components and the Hybrid Aerogel

The thermal stability of the aerogel was investigated by TGA since it is important to establish the temperature sensitivity of the formed hybrid structure. The differential thermogravimetric (DTG) curves of the hybrid aerogel and its components are shown in Figure S4. All samples showed an initial weight loss while heated from 40 to 120 °C due to moisture evaporation, followed by a sharp weight loss due to thermal degradation of cellulose and the amyloid peptide. The onset of the thermal degradation for DA-C-CNFs and ANFs was 197 and 213 °C, respectively, while in the hybrid aerogels, thermal decomposition began at a higher temperature (approximately 241 °C). Moreover, a similar trend was also observed for the DTG peak temperatures, which shifted from 320 °C for DA-C-CNFs and 330 °C for ANFs to 340 °C for the hybrid system. This shows that the thermal stability of the hybrid system increased compared to its individual components. The detailed explanation for this increased thermal stability is not known, but it has been shown that the type of counterion to the CNFs has a significant effect on the thermal stability of the CNFs.^51^ In our hybrid system, the charges of the ANF and CNF are acting as counterions to each other, meaning that the low-molecular-weight counterions are released, and this might explain the improvement in the thermal properties of the hybrid aerogel.

### 3.4 Adsorption of Dyes and Metal Ions at Different pHs

**Figure 4:**
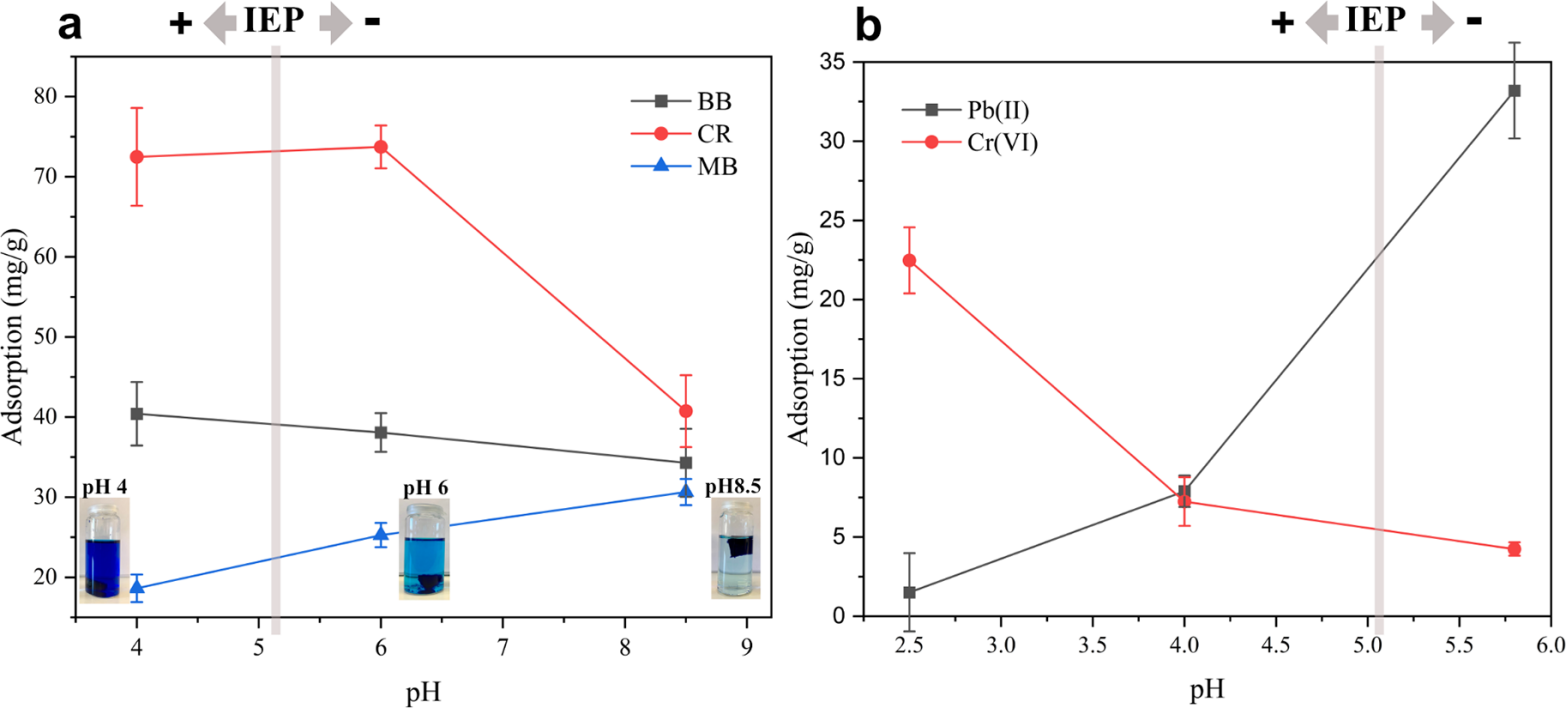
Adsorption capacity of DA-C-CNF/ANF aerogels as a function of pH for (a) dyes with photographs of MB adsorption experiments taken 48 h after submersion and (b) heavy metals. IEP refers to the isoelectric point of ANFs.

To demonstrate the versatility and potential use of our hybrid aerogels in water remediation applications, we carried out both metal ion and contaminant dye adsorption studies. The pH of the solution has a significant effect on the adsorption of organic dyes and metal ions due to changes in the degree of ionization and state of solution of the contaminants and the adsorbents.^52^ Under the range of pHs studied here, CR and BB predominantly existed in the anionic form owing to the ionization of sulfonated functional groups, while MB was predominantly in the cationic form. As shown in Figure 4a, changes to the pH impacts adsorption, with the adsorption of anionic dyes decreasing with increasing pH while the adsorption of the cationic dye is increasing with increasing pH.

The binding of dyes to the aerogels occurs due to three possible reasons: (i) entropy gain due to the release of counterions to the charged groups on the hybrid aerogels and the dyes;^53^ (ii) entropy gain due to the release of water molecules on the hybrid aerogels and the dyes, also known as hydrophobic interactions;^54,55^ and (iii) polar interactions between the polar groups of dyes and the aerogel.^22^ Most likely, these interactions are also of entropic origin. The degree of contribution of each mechanism is different for each dye and is highly affected by the pH of the solution at a constant temperature.

To examine the contribution from the counterion release, the zeta potential of the aerogel components was measured at different pH values. As shown in Figure 1b, DA-C-CNFs are negatively charged due to the ionization of carboxylic acid groups, with the charge density increasing with pH. Comparatively, ANFs are positively charged at low pH values and negatively charged at higher pHs with an isoelectric point of around 5.2, which is in the same range as earlier reported values for β-lactoglobulin derived ANFs.^56,57^

Due to the oppositely charged ANFs and DA-C-CNFs, the hybrid aerogel will have an amphoteric nature that will give an interaction with both anionic and cationic components over a rather wide pH range. However, at pHs larger than 5.2, the aerogels will have a net negative charge, and adsorption of BB and CR will decrease due to the electrostatic repulsions between these dyes and the negatively charged aerogel. However, still there is significant adsorption at pH 6.0 and 8.5, especially for BB, indicating that charge-driven interactions, i.e., the entropy gain due to the release of counterions, are not solely responsible for the interactions, in turn implicating that hydrophobic interactions, i.e., the release of water molecules, play a significant role for the adsorption. Moreover, localized interactions possibly occur between the polar groups of dyes and amino acid residues on the ANFs or hydroxyl groups of DA-C-CNFs, but these are most likely very short range and only occur once the molecules are already at the interface. However, molecular simulations on the binding of CR on ANFs suggest that CR binds to the sites on the fibril surface antiparallel to the β-sheets.^58^ Nevertheless, it is not possible to specify which amino acid residues on the ANFs surface are interacting with the dyes since the detailed molecular structure of ANFs is still unknown.^22,59^ MB, on the other hand, adsorbed the least at pH 4.0 since the intensified charge-driven repulsions between positively charged ANFs and MB hinder the adsorption. At pH 8.5, both ANFs and DA-C-CNFs are negatively charged, making the adsorption of positively charged MB favorable.

In addition to the organic dyes, the adsorption capacity of the aerogel for Cr(VI) and Pb(II) was evaluated within the pH range of 2.5 to 5.8 (Figure 4b). Adsorption of Cr(VI) was the highest at pH 2.5 and decreased rapidly when the pH was increased. Within the pH range of 2.5–5.8, chromium ions predominantly exist in the form of hydrogen chromate (HCrO^4–^) and dichromate (Cr_2_O_7_^2–^).^60^ Hence, the optimal adsorption capacity of the aerogel is at pH 2.5 due to the high net charge of the aerogels at this pH where multivalent dichromate ions will show a much higher affinity to the aerogel than their original monovalent counterions. At pH 2.5, the zeta potential of the ANFs exceeded 48 mV (Figure 1b) due to the protonation of the abundant amine groups, with Cl^–^ as the counterion, which results in an efficient counterion exchange with the chromium oxyanions. Furthermore, at this pH, the carboxylic acid groups of DA-C-CNFs are protonated, and the possible repulsion forces between DA-C-CNFs and anionic chromium ions are at a minimum. When the pH increased, carboxylic acid groups are gradually deprotonated and amine groups are also deprotonated, which hinder the adsorption of negatively charged chromium species. Adsorption of lead, on the other hand, follows the opposite trend. Lead exists in the form of Pb2+ ions within the studied pH range.^61^ Thus, the Pb(II) binding is maximized over a pH of 5.8 where both ANFs and DA-C-CNFs have a negative zeta potential and then reduces drastically at decreasing pHs. This is attributed to the protonation of the amine functionalities of ANFs that leads to the formation of electrostatic repulsion forces; i.e., the Pb(II) will have negative adsorption to the cationic surfaces. A very similar trend has been reported for the adsorption of lead by amyloid lysozyme fibrils conjugated with polyethyleneimine.^62^

These results demonstrated that by tuning the surface charge of the aerogels via a rather simple tuning of the pH of the solution, the wet-stable, amphoteric aerogels can be utilized for the removal of both cationic and anionic contaminants.

### 3.5 Adsorption Isotherms

**Figure 5:**
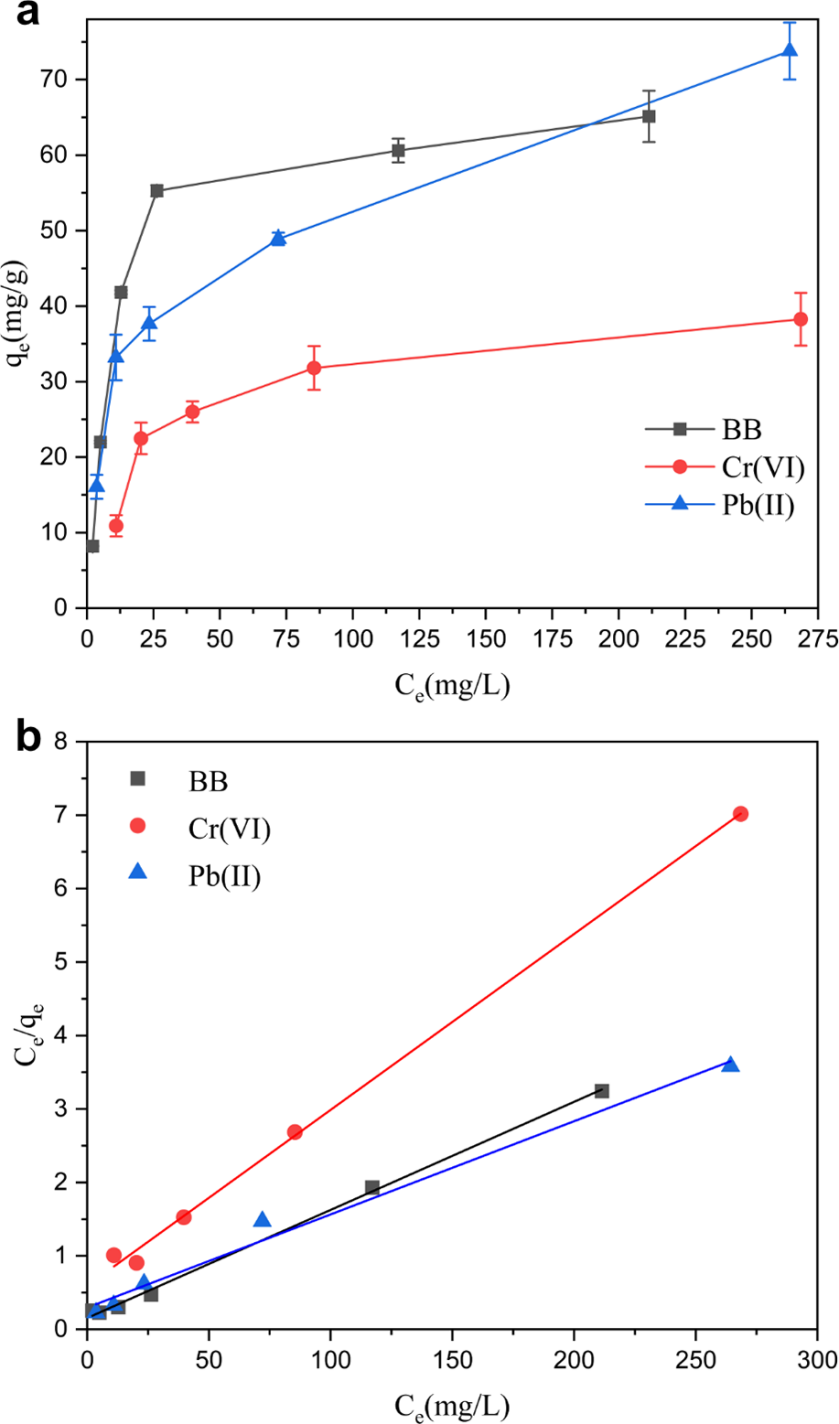
(a) Adsorption isotherms of BB at pH 4, Cr(VI) at pH 2.5, and Pb(II) at pH 5.8 onto the aerogel (adsorption tests were performed at the optimum pH for each contaminant). (b) Adsorption data fitted to the Langmuir model.

Metal ions and BB were chosen as model contaminants to further investigate the interactions and determine the maximum saturation adsorption capacity of the hybrid aerogels. The adsorption isotherm curves of BB, Cr(VI), and Pb(II) are shown in Figure 5a. It is noted that the equilibrium adsorption capacity increased with the equilibrium concentration of the solution until it reached a plateau (except for Pb(II)). Moreover, Langmuir (Figure 5b) and Freundlich models (Figure S5) were employed to analyze the experimental data, and the model parameters are shown in Table 2. A comparison of the correlation coefficient of Langmuir (*R*^2^ ∼0.99 for all sorbates) and Freundlich models (*R*^2^ in the range of 0.79 to 0.93) demonstrates that the Langmuir model shows a good description of the adsorption for the contaminants onto the DA-C-CNF/ANF aerogels. As is well known, the Langmuir model suggests that the adsorption occurs as a monolayer and that all the adsorption sites on the surface of aerogel are equivalent with uniform binding affinity.^63^ Moreover, the good fit to the Langmuir model suggests that there is no interaction between the adsorbate molecules; thus, the adsorption is independent of whether the neighboring sites are occupied or not.^64^ The theoretical maximum adsorption capacity of DA-C-CNF/ANF aerogels for BB, Cr(VI), and Pb(II) was calculated to be 67.9, 41.8, and 78.8 mg g^–1^, respectively.

**Table 2:** Parameters of the Langmuir and the Freundlich Isotherms Models

		adsorbate		
isotherm model	parameter	BB	Pb(II)	Cr(VI)
Langmuir	*R*^2^ (%)	0.998	0.987	0.998
	*q*_max_ (mg/g)	67.93	78.80	41.77
	*K*_L_ (L/mg)	0.095	0.043	0.040
Freundlich	*R*^2^ (%)	0.791	0.939	0.811
	*K*_F_ (L/g)	10.112	12.501	6.304
	*n*	2.491	3.050	2.874

### 3.6 Adsorption Kinetics

**Figure 6:**
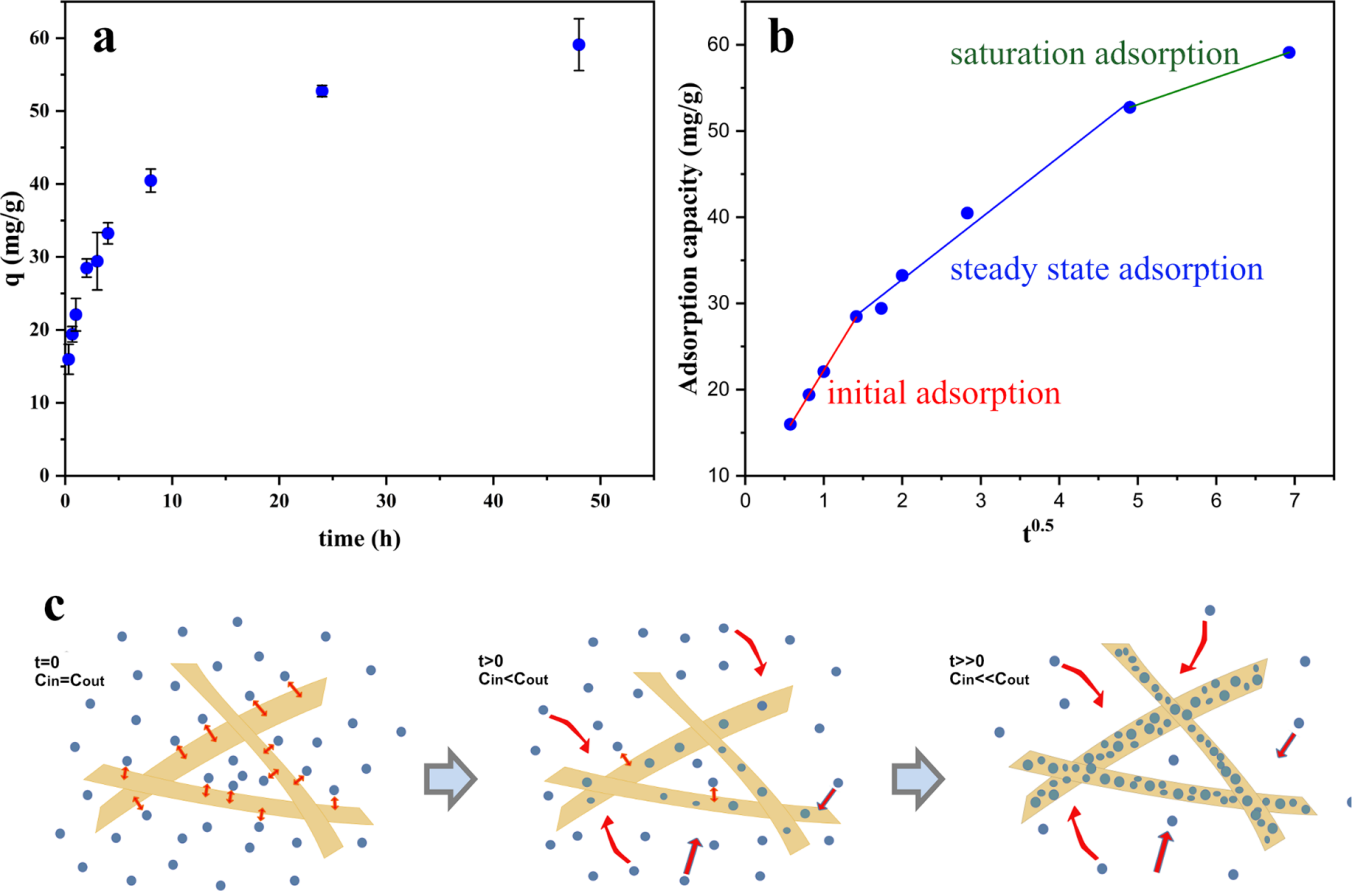
(a) Adsorption kinetics of BB on DA-C-CNF/ANF aerogels at pH 4. (b) Kinetics data fitted to the intraparticle diffusion model. (c) Schematic representation of each step in the intraparticle diffusion model.

To further elucidate the adsorption process of the pollutants in our hybrid aerogels, the adsorption of BB was investigated over time. Figure 6a shows that the adsorption capacity increased sharply within the first 8 h and then leveled off gradually. Experimental data were fitted to a pseudo-first-order and pseudo-second-order model (Figure S6). The results indicate that the pseudo-second-order model better describes the adsorption kinetics of BB in the aerogel, with an *R*^2^ ∼0.99 and good agreement between the experimental *q*_e(exp)_ and the calculated *q*_e(cal)_ values (Table S1). In a simple case, the pseudo-second-order model assumes that the rate-limiting step in the adsorption process is chemisorption, but it could also indicate coupled processes in the adsorption process.^63,65^

To further clarify the processes and to determine whether the intraparticle diffusion plays a significant role in the adsorption rate of BB in the aerogel, the intraparticle diffusion model was tested, and the results are shown in Figure 6b. The plot of *q*_t_ vs *t*^0.5^ shows three linear regions during the adsorption process, indicating that although intraparticle diffusion does play role in the adsorption, it is not the sole rate-limiting step.^66^ This is displayed schematically in Figure 6c where the first sharp step is attributed to the adsorption of the adsorbate to the external layer of the adsorbent (initial adsorption). During the adsorption, the dye concentration in the solution inside the aerogel pores decreases, and the dyes gradually diffuse from the surrounding media into the pores of the aerogel. In this step, intraparticle diffusion is rate-limiting, and we achieve steady-state adsorption. In the last step, the concentration of the adsorbate in the solution is low, and the adsorbent is partially saturated; hence, the adsorption rate slows down (saturation adsorption).^67^ This latter matching of the data shows that the adsorption process is most probably a diffusion limited process that leads to an apparent pseudo-second-order model.

### 3.7 Adsorbent Regeneration

**Figure 7:**
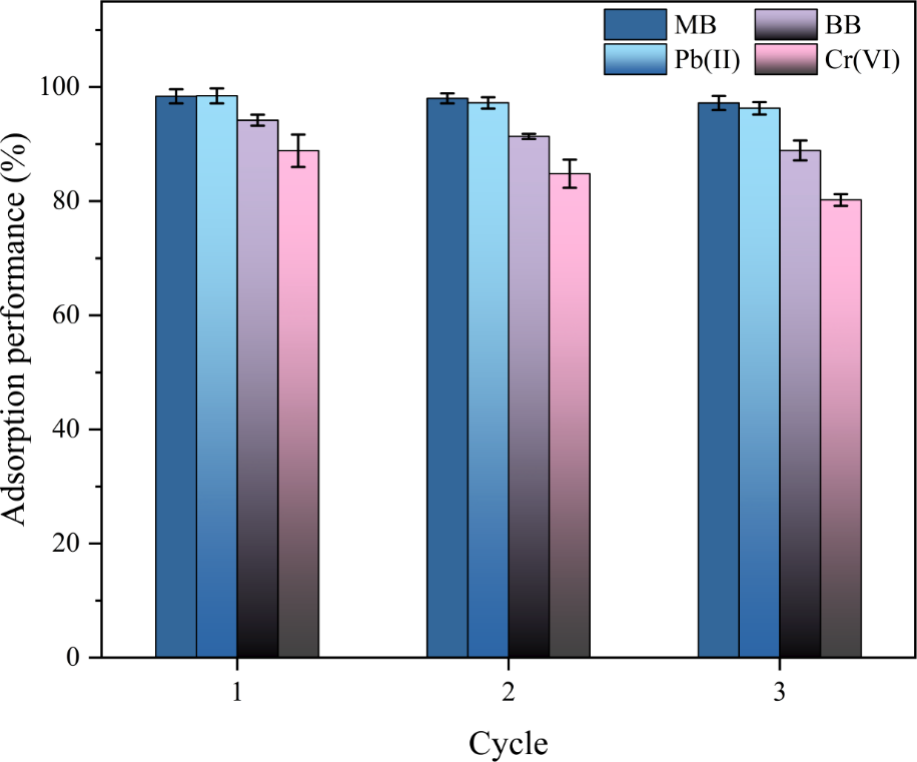
Adsorption performance of the composite aerogel over three regeneration cycles for MB, Pb(II), BB, and Cr(VI).

The reusability of an adsorbent is a crucial economic and environmental factor for water treatment. Hence, the reusability of the DA-C-CNF/ANF aerogels for the adsorption of dyes and heavy metals was tested by performing cyclic adsorption/desorption tests. After adsorption, the BB and Cr(VI)-saturated aerogels were washed with 0.01 M NaOH solution, while the MB and Pb(II)-saturated aerogels were washed with 0.01 M HCl solution to elute the adsorbates. From this, the aerogels were re-exposed to the model contaminates, and the adsorption was measured. As shown in Figure 7, that aerogels maintained nearly 97 and 96% of their adsorption capacity for MB and Pb(II) as cationic moieties after three cycles of regeneration, while for the anionic moieties, the adsorption capacity of the aerogels decreased gradually after each desorption cycle in alkaline media. The adsorption capacity of the aerogel for BB and Cr(VI) after the third regeneration cycle was 89 and 80% of the initial adsorption capacity. While the adsorption of anionic compounds after three cycles is lower than that of cationic compounds, it is still highly efficient and shows their potential as suitable adsorbents for practical water treatment processes.

The adsorption capacity, reusability, and preparation method of recently reported amyloid-based adsorbents for the removal of Pb(II) are compared in Table 3. The adsorption capacity of the DA-C-CNF/ANF aerogels is in the same range as plant-based amyloid membranes but lower than freeze-dried amyloid cross-linked aerogels, amyloids in particle form, and individual amyloid nanofibrils. However, DA-C-CNF/ANF aerogels are prepared via a scalable and more cost- and energy-efficient technique since they do not require freeze-drying.^68^ Moreover, they can be easily regenerated under rather mild acidic conditions and reused, maintaining 96% of their adsorption capacity after the third cycle, which is considerably higher than most of the earlier reported values, especially that of amyloid particulates that kept only 29% of their adsorption capacity after four cycles^69^ and individual β-lactoglobulin nanofibrils that retained only 85% of their adsorption capacity after one cycle.^70^ Besides, implementation and regeneration of aerogels are easier than individual nanofibrils and particulates. Thus, considering their facile and green preparation process, ease of regeneration, reusability, and the pH tunable surface charge that enables adsorption of both cationic and anionic contaminants including a variety of organic dyes and metal ions, the amphoteric DA-C-CNF/ANF aerogels are promising sustainable adsorbents for water purification.

**Table 3:** Summary of Recent Literature on Amyloid-Based Adsorbents for Removal of Pb(II)^a^

adsorbent	*q*_e_ Pb(II) (mg/g)	preparation method	regeneration (condition/cycles/adsorption performance remained in the last cycle)	reference
sun flower amyloid fibril membranes	87.3	vacuum filtration		(71)
peanut amyloid fibril membranes	72.3	vacuum filtration		(71)
polydopamine coated nanocellulose/amyloid aerogel	17.82	freeze drying	3 cycles using HNO_3_ pH 3, 83%	(72)
amyloid particulates from BSA	211.3	freeze drying	4 cycles using 0.1 M EDTA solution, 29%	(69)
amyloid cross-linked aerogels	161.1	freeze drying		(24)
amyloid/ZIF-8 hybrid aerogel	318	freeze drying	5 cycles using 0.1 M EDTA solution, 76%	(24)
bovine serum albumin (BSA) nanofibrils based biofilms	110.39	reverse osmosis		(73)
amyloid-fibrils-like functional materials	11.5	fibrils were used in suspension form		(74)
β-lactoglobulin nanofibrils	212	freeze drying	1 cycle using 2% HCl solution, 85%	(70)
polydopamine modified lysozyme amyloid-CNF membranes	270.3	vacuum filtration	5 cycles using 1% HCl + 5% Ca(NO_3_)_2_, 85%	(75)
**DA-C-CNF/ANF aerogels**	**78.8**	**ambient dried**	**3 cycles using 0.01 M HCl, 96%**	**current work**

a The table lists the maximum adsorption capacity, preparation method, regeneration condition, and adsorption performance of the aerogel after the last regeneration cycle.

### 3.8 Selectivity

**Figure 8:**
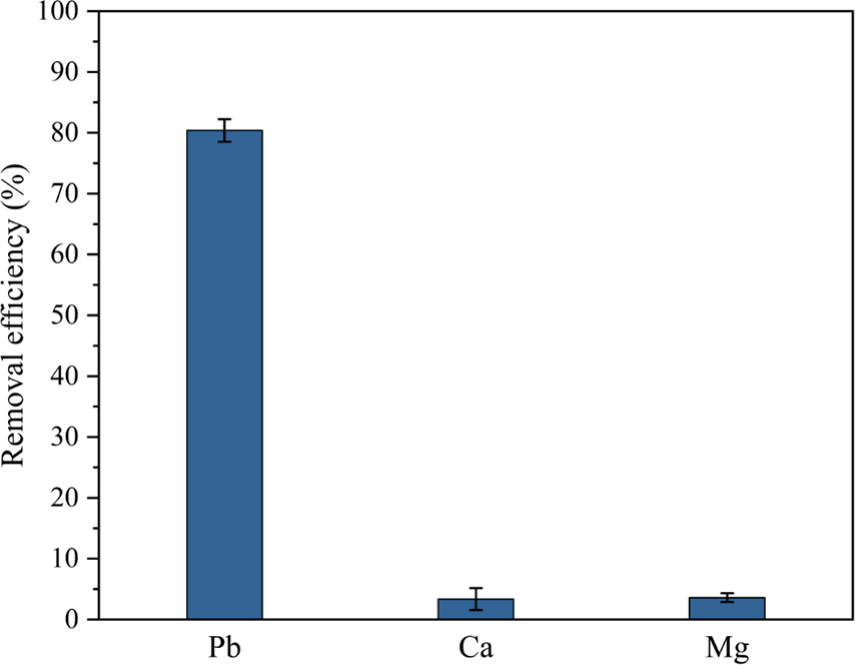
Removal efficiency of aerogels for Pb2+, Ca2+, and Mg2+ in tertiary ion-mixed solutions (initial concentration: 14 mg L^–1^ of each ion; adsorbent dosage: 0.53 g L^–1^.

Natural waters such as lakes and rivers contain a remarkable amount of calcium and magnesium ions that can negatively affect the adsorption performance of the aerogels, for example, for lead ions. In this respect, the ion selectivity of the aerogels toward Pb^2+^ in the presence of competing Ca^2+^ and Mg^2+^ ions was evaluated in an ion-mixed solution. Figure 8 shows that lead was indeed selectively adsorbed by the aerogel without any significant interference from the background ions. It has been widely recognized that the hydrated ionic radius and electronegativity of metal ions have a substantial influence on their adsorption performance onto different adsorbents.^76^ The higher electronegativity of Pb^2+^ (Table 4) compared to Ca^2+^ and Mg^2+^ ions and its lower hydrated radius most probably lead to the higher affinity of Pb^2+^ to the aerogel surface. Hence, in a multicomponent ion solution, Pb^2+^ ions preferentially adsorb on the surface and occupy the adsorption sites, leading to the lower uptake efficiency for other metal ions.^77^ To further quantify the selective adsorption of lead, the ion distribution coefficient (*K*_d_) and selectivity coefficient (α) were calculated (Table 4). The aerogels exhibit a *K*_d_ value of 7562 for lead ions, which is more than 2 orders of magnitude higher than those for divalent background metal ions, demonstrating a remarkable selectivity for Pb^2+^.

**Table 4:** Physiochemical Properties of Metal Ions and Selective Adsorption Parameters of Aerogels toward These Ions

metal ion	electronegativity (Allen scale)	hydrated radius (Å)	*K*_d_ (mL/g)	α^a^
Pb^2+^	1.85	4.01	7562 ± 931	
Ca^2+^	1.03	4.1	65 ± 36	116.7
Mg^2+^	1.29	4.28	70 ± 29	107.9

a α is the ratio between *K*_dPb_ and *K*_d_ of the background ion.

## 4 Conclusions

Effective aerogel adsorbents based on sustainable DA-C-CNFs and ANFs were prepared via an energy-efficient and scalable freeze-linking technique that facilitated the formation of highly porous aerogels using ambient drying conditions. The formation of the porous structure relied on ice templating in which the growth of ice crystals forced the DA-C-CNF/ANFs into lamellae between the ice crystals. Meanwhile, this dramatic decrease in the distance between the fibrils also allows for the formation of hemiacetal linkages between DA-C-CNFs, which inherently will cross-link the entire structure and physically lock the ANFs in a wet resilient structure. The frozen network was then thawed, solvent exchanged, and dried under ambient conditions, resulting in low densities (18–28 kg m^–3^), high porosity, and mechanical robustness in both the wet and dry states. Microstructure characterization by SEM and APVD revealed that the pores were polydisperse in size with the majority having a diameter of approximately 50 μm. Confocal microscopy showed that ANFs were uniformly dispersed in the structure although associated with the DA-C-CNFs due to the interaction between the oppositely charged fibrils. Owing to the pH-tunable surface charge of the aerogel, it could efficiently adsorb both cationic and anionic species by simply adjusting the pH. Adsorption analysis revealed that adsorption followed the Langmuir model, and the saturation adsorption capacity for BB, Cr(VI), and Pb(II) was 67.9, 41.8, and 78.8 mg g^–1^, respectively. The kinetics of sorption of BB as a model dye was investigated and fit well to a pseudo-second-order model, but this is most probably due to a diffusion limited process that leads to an apparent second-order model. After adsorbing contaminants, the aerogels were easily regenerated and reused for at least three cycles, maintaining 97 and 96% of their adsorption capacity for MB and Pb(II) as cationic moieties and 89 and 80% of their initial adsorption capacity for Cr(VI) and BB as anionic model contaminants. Finally, the selectivity toward Pb(II) in the presence of calcium and magnesium ions was tested, and the aerogel showed remarkable selectivity for Pb(II). Overall, the use of sustainable materials with energy-efficient production, versatile adsorption behavior (anionic and cationic speciation), easy and efficient regeneration, and good selectivity for Pb(II) makes hybrid DA-C-CNF/ANF aerogels promising materials for water treatment applications.

## Supporting Information Available

Calibration curves of dyes at different pHs; AFM images of ANFs; densities of aerogels prepared using different initial concentrations; TGA and DTG curves of DA-C-CNF, ANF, and biohybrid aerogel; adsorption data of BB, Cr(VI), and Pb(II) fitted to the Freundlich model; and pseudo-first-order and pseudo-second-order model fitted to the experimental data of adsorption of BB on the aerogels (PDF)
