# Supplementary material for: Green Ambient-Dried Aerogels with a Facile pH-Tunable Surface Charge for Adsorption of Cationic and Anionic Contaminants with High Selectivity
Source: Biomacromolecules. 2022 Nov 1;23(11):4934–47. doi: 10.1021/acs.biomac.2c01142 (PMC9667464; doi:10.1021/acs.biomac.2c01142)
Supplement: Supplementary file 1 — bm2c01142_si_001.pdf [file bm2c01142_si_001.pdf]

Green ambient-dried aerogels with a facile pH-tunable surface charge for adsorption of cationic and anionic contaminants with high selectivity

Zhaleh Atoufi [a], Goksu Cinar Ciftci [a], Michael S. Reid [a], Per A. Larsson [a], Lars Wågberg[a,b*]*

[a] Department of Fiber and Polymer Technology, KTH Royal Institute of Technology, Teknikringen 56–58, SE-100 44 Stockholm, Sweden.

[b] KTH Royal Institute of Technology, Department of Fiber and Polymer Technology, Wallenberg Wood Science Center (WWSC), Stockholm, Sweden

[*] Corresponding authors: zhaato@kth.se, Tel: +46-764556445, wagberg@kth.se, Tel: +46-87908294

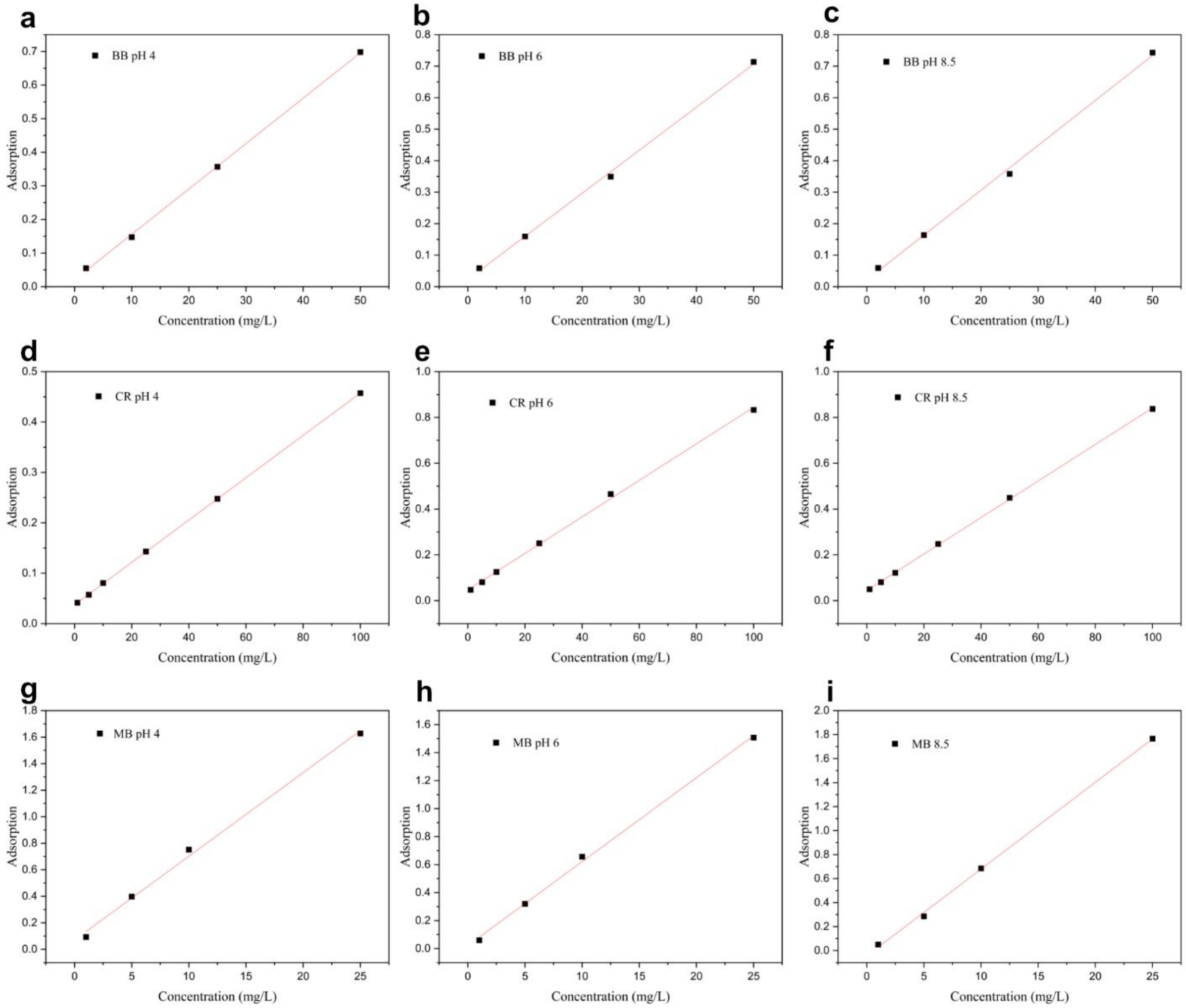

Figure S1. Calibration curves of dyes. Adsorption of BB at a) pH 4, b) pH 6, c) pH 8.5 and CR at d) pH 4, e) pH 6, f) pH 8.5, and MB at g) pH 4, h) pH 6, and i) pH 8.5 within their linier region.

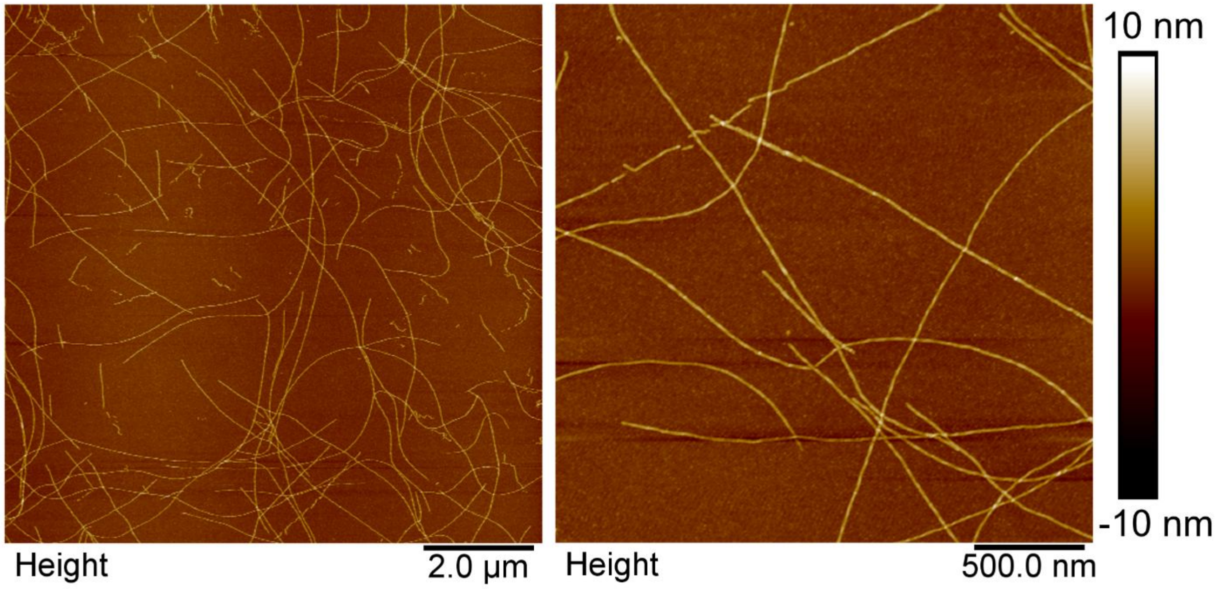

Figure S2. AFM images of ANFs with two different magnifications.

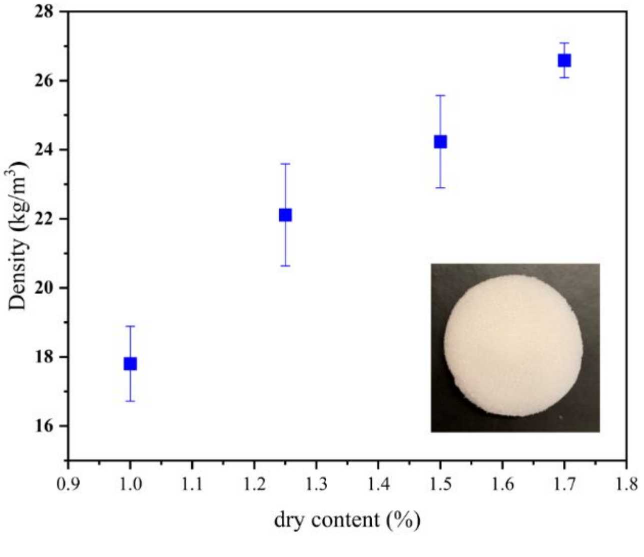

Figure S3. Densities of aerogels prepared using various initial concentrations; the inset is the picture of the aerogel with lowest density.

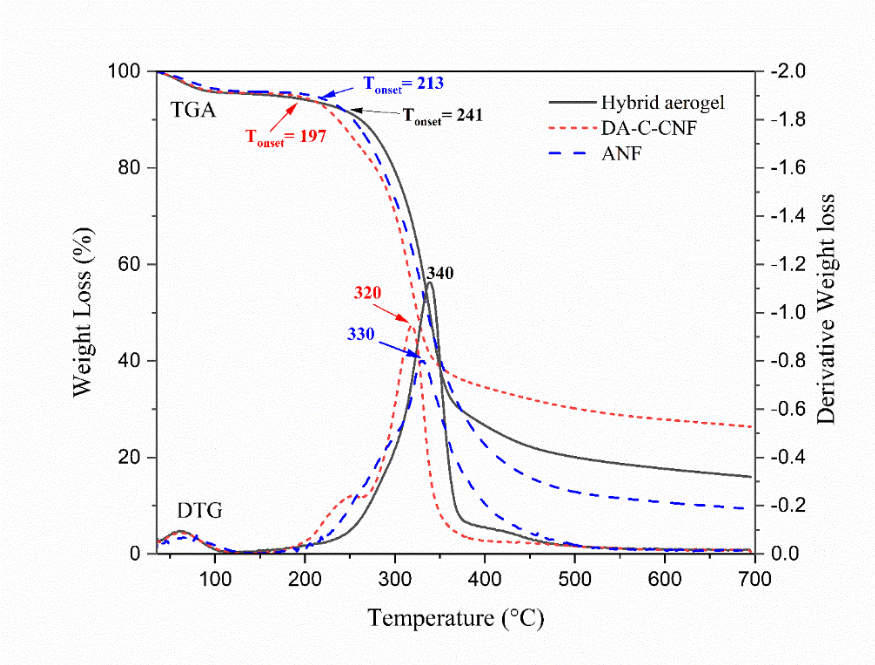

Figure S4. TGA and DTG curves of DA-C-CNF, ANF, and the biohybrid aerogel.

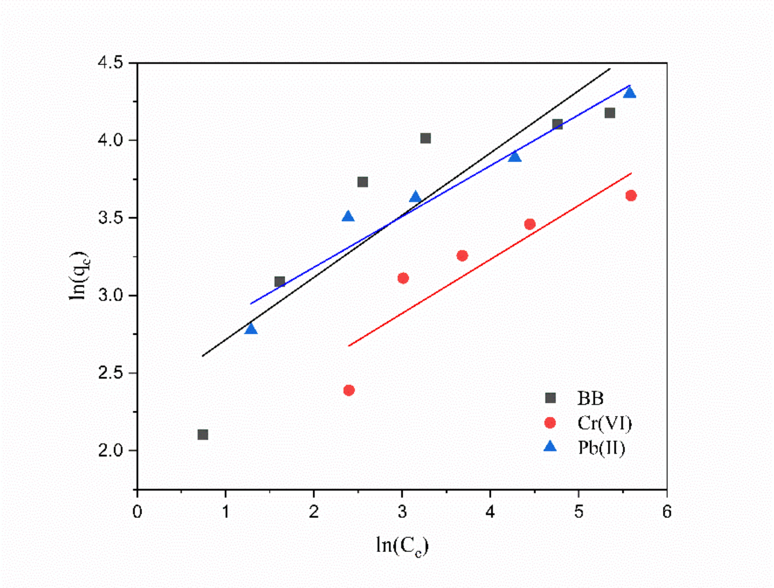

Figure S5. Adsorption data of BB, Cr(Ⅵ) and Pb(Ⅱ) fitted to Freundlich model.

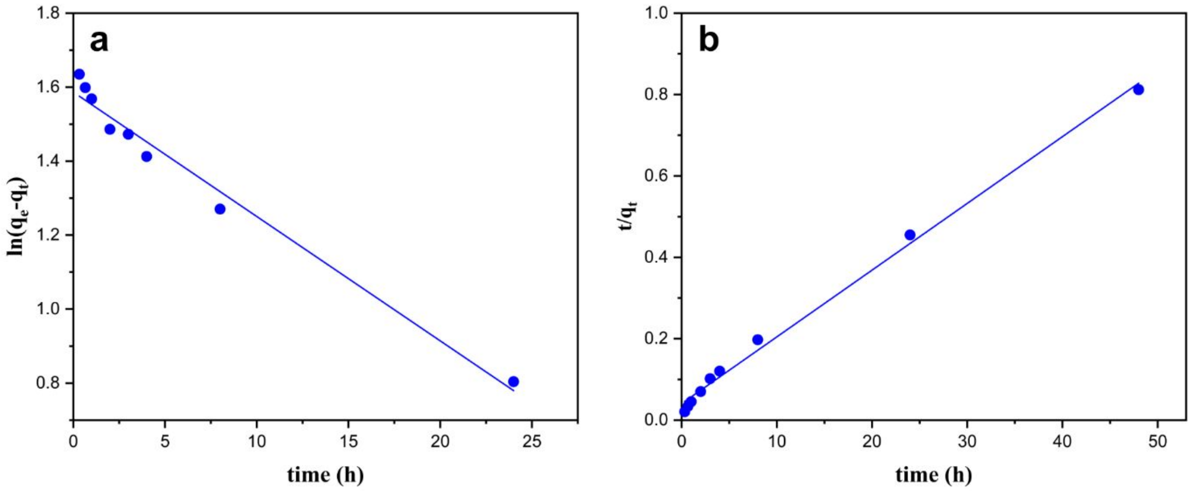

Figure S6. Pseudo-first-order and Pseudo-second-order model fitted to the experimental data of adsorption of BB on the aerogels.

Table S1. Pseudo-first-order and Pseudo-second-order parameters for the adsorption of BB on the DA-C-CNF/ANF aerogel.

Kinetic Model	K_1	K_2	$q_{e\ exp}$	$q_{e\ cal}$	R^2
Pseudo first order	0.0336	-	59.116	4.888	0.979
Pseudo second order	-	0.007	59.116	60.97	0.995
